# Prevalence of hyperemesis gravidarum and its associated electrolyte and hematologic disturbances in pregnant women

**DOI:** 10.3389/fmed.2026.1703120

**Published:** 2026-02-23

**Authors:** Heba Bassiony Ghanem, Shaher Mustafa Hammoudeh, Moaz Abulfaraj, Dalia Mahmoud Abdelmonem Elsherbini, Sanaa Elfatih Hussein, Jamila Ibrahim Algader, Raghad Mohammed Alanazi, Taif Yahya Alshammari, Amirah Enad Alruwaili, Najd Budayri Alruwaili, Mohamed El-Sherbiny, Ahmed Baker A. Alshaikh, Maged Elshamy, Mohamed Mahmoud Abdelfattah Abdelrahman, Nagwan Ahmed Bahgat

**Affiliations:** 1 Department of Clinical Laboratory Sciences, College of Applied Medical Sciences, Jouf University, Sakaka, Saudi Arabia; 2 Department of Pediatric and Neonatology, El Sharq Hospital, Fujairah, United Arab Emirates; 3 Department of Surgery, Faculty of Medicine, King Abdulaziz University, Jeddah, Saudi Arabia; 4 Department of Basic Medical Sciences, College of Medicine, AlMaarefa University, Riyadh, Saudi Arabia; 5 Research Center, Deanship of Scientific Research and Post-Graduate Studies, AlMaarefa University, Riyadh, Saudi Arabia; 6 Department of Obstetrics and Gynecology, College of Medicine, Jouf University, Sakaka, Saudi Arabia; 7 Department of Anesthesia, Surgical Intensive Care and Pain Management, Faculty of Medicine, Mansoura University, Mansoura, Egypt; 8 Department of Anesthesia and Critical Care, King Abdulaziz University Hospital, King Abdulaziz University, Jeddah, Saudi Arabia; 9 Department of Obstetrics and Gynecology, Faculty of Medicine, Mansoura University, Mansoura, Egypt

**Keywords:** Aljouf region, electrolyte disturbance, hematological disturbance, hyperemesis gravidarum, Maternity care, pregnancy

## Abstract

**Background and objective:**

Hyperemesis gravidarum (HG) is a condition that develops early in pregnancy, before 16 weeks of gestation, and is characterized by severe nausea and/or vomiting, inability to tolerate food and/or beverages, and substantial impairment of daily activities. Hyperemesis gravidarum can have obvious maternal and fetal consequences. This study aimed to assess the prevalence of HG, investigate its associated electrolyte and hematologic disturbances, and identify risk factors among pregnant women in Aljouf, Saudi Arabia.

**Methods:**

A retrospective observational case-control study was conducted using 200 medical records selected from a total of 9,090 records of pregnant women at the Maternity and Children Hospital in Aljouf, covering the period from November 2020 to November 2023.

**Results:**

The prevalence of hyperemesis gravidarum was 1.1% among the studied pregnant women. Patients diagnosed with hyperemesis gravidarum showed significantly lower body weight and BMI than controls. Nearly 48% of HG cases occurred in women aged 30–39 years, while 52% were in the first trimester. Furthermore, 36% of the affected women were primigravida, and 11% were pregnant with twins. Hypotension was observed in 35% of HG cases. Hyponatremia occurred in 29% of patients, hypokalemia in 21%, and hypochloremia in 24%. Hematological disturbances included increased Hb levels in 11% of cases, increased hematocrit in 12%, leukocytosis in 19%, and neutrophilia in 17% of the patients.

**Conclusion:**

Hypotension, electrolyte imbalance, and hematological disturbances are the primary consequences of HG. First-trimester pregnancy and low gestational age are the most important risk factors for HG. Strategies by healthcare centers and further research are needed to enhance the treatment, management, and prevention methods of HG.

## 1 Introduction

Nausea and vomiting in pregnancy (NVP) are prevalent conditions, affecting approximately 80% of pregnant women to varying degrees (1). Severe or prolonged NVP is commonly termed as hyperemesis gravidarum (HG), impacting approximately 0.30 to 3.6% of pregnancies (2). Hyperemesis gravidarum (HG) is a condition that begins early in pregnancy, before 16 weeks of gestation, and is marked by intense nausea and/or vomiting, inability to consume food and/or liquids normally, and significant restriction on daily activities. Dehydration indicators are considered key factors in diagnosing hyperemesis gravidarum (3).

HG is the most frequent reason for hospitalization in the early half of pregnancy, second only to premature labor across the entire gestational period. It can cause potentially life-threatening pregnancy complications (4, 5) due to substantial weight loss from insufficient caloric intake, along with dehydration and electrolyte imbalance. Rarely, vitamin deficiencies can cause severe maternal morbidity or death, including thiamine deficiency, which may lead to Wernicke encephalopathy (6).

The development of HG has been linked to the placenta and the appetite-regulating hormone gene growth differentiation factor 15 (GDF15) (7). GDF-15 binds to receptors in the brain’s “vomiting center” located in the postrema area, stimulating this center and inducing vertigo, nausea, and vomiting. These symptoms have been found to be closely associated with HG (8, 9).

Potassium homeostasis generally remains stable during pregnancy despite numerous physiological alterations that favor renal potassium excretion, including an increase in blood volume, enhanced renal perfusion, elevated glomerular filtration rate (GFR), and activation of the renin-angiotensin-aldosterone system. Serum potassium concentrations are maintained within physiological ranges through elevated levels of progesterone, which inhibits kaliuresis. This compensatory mechanism is susceptible to rapid exhaustion caused by HG, which may result in significant electrolyte disturbances and encephalopathy (10). Approximately 40% of women with HG experience hypokalemia, whereas 36 and 8% exhibit hyponatremia and hypochloremia, respectively (11).

Nutritional deficits resulting from hyperemesis gravidarum may lead to maternal and fetal complications. Potential effects may include weight loss, dehydration, muscular weakness, anxiety, depression, and post-traumatic stress disorder during pregnancy. All of these issues have been linked to negative pregnancy outcomes for the fetus, such as preterm births (PTBs), low birth weight (LBW), small for gestational age (SGA), and low 5-min Apgar scores (12).

The health system needs to be strengthened to enhance women’s and children’s health, lower mortality rates, and encourage safe pregnancies (13). Identifying the underlying factors of HG and implementing early intervention may reduce the negative perinatal outcomes, hospitalization rates, and physical, psychological, economic, and social burden of HG in women’s lives (4). Therefore, the present study aimed to assess the prevalence of HG, the associated electrolyte and hematologic disturbances, and the risk factors among pregnant women in the Aljouf Region, Saudi Arabia.

## 2 Subjects and methods

### 2.1 Study design and duration

A retrospective observational case-control study was conducted to estimate the prevalence of hyperemesis gravidarum and to compare clinical characteristics between HG-affected and non-affected (control) pregnant women. This study was approved by the Research Ethics Committee of Qurayyat Health Affairs (IRB Approval No. 2023-111), Saudi Arabia, and the data were kept in strict confidentiality.

The collected data were obtained from 200 medical records selected from 9,090 medical records of pregnant women (before 20 weeks of gestation) who attended antenatal care visits or were admitted to the Maternity and Children Hospital in Sakaka, Aljouf, Saudi Arabia, between November 2020 and November 2023.

#### 2.1.1 Study groups

Group I (control group) included pregnant women before 20 weeks of gestation who visited antenatal care centers at the study hospitals and did not have HG.

Group II (HG group) included pregnant women before 20 weeks of gestation who were admitted to the hospital and diagnosed with HG.

The diagnostic criteria for hyperemesis gravidarum (HG) in the hospital depend mainly on clinical presentations, such as severe vomiting (≥3 times per day), severe nausea affecting normal consumption of food and/or liquids, and weight loss (≥5% of body weight).

The inclusion criteria for the selected cases were medical records of pregnant women (up to 20 weeks of gestation) with a documented diagnosis of hyperemesis gravidarum (HG). For the selected controls, the criteria included pregnant women who sought antenatal care services (before 20 weeks of gestation) without a documented diagnosis of HG. The study population for both the case and control groups comprised exclusively pregnant women residing in the Al-Jouf region who possessed comprehensive medical records necessary for data analysis.

Additionally, the exclusion criteria included pregnant women beyond 20 weeks of gestation, those residing outside the Al-Jouf region, individuals with chronic conditions such as diabetes mellitus, hypertension, renal diseases, liver diseases, thyroid disorders, and gastric or intestinal diseases, and individuals with incomplete medical records necessary for analysis.

### 2.2 Sample size

The adequacy of the sample size (200 medical records, including 100 HG cases that were detected from 9,090 medical records and compared with 100 controls from the total collected reports) was ensured, taking into account the Al-Jouf population that was approximately 595,822 in 2023. The expected incidence of HG among pregnancies ranges from 0.3 to 3.6% of pregnancies (2), as calculated using a formula available on a relevant website: https://www.calculator.net/sample-size.

### 2.3 Data analysis

Demographic, obstetric, and clinical data (age, body weight, BMI, gravidity, gestational age, type of gestation (single or multiple), previous abortion, and previous history of HG) were analyzed.

Complete blood count and serum electrolyte levels were analyzed using SPSS (version 23) statistical software. The results were analyzed using the chi-squared test, Fisher’s exact test, and independent *t*-test. To identify high-risk variables associated with HG, a binary logistic regression analysis was conducted to calculate odds ratios with 95% confidence intervals and *p*-values in order to assess the association between dependent and independent variables. A *p*-value of <0.2 was used for the multivariable logistic regression test. Differences were considered statistically significant at *p* < 0.05.

## 3 Results

### 3.1 Prevalence of hyperemesis gravidarum cases

**Figure 1:**
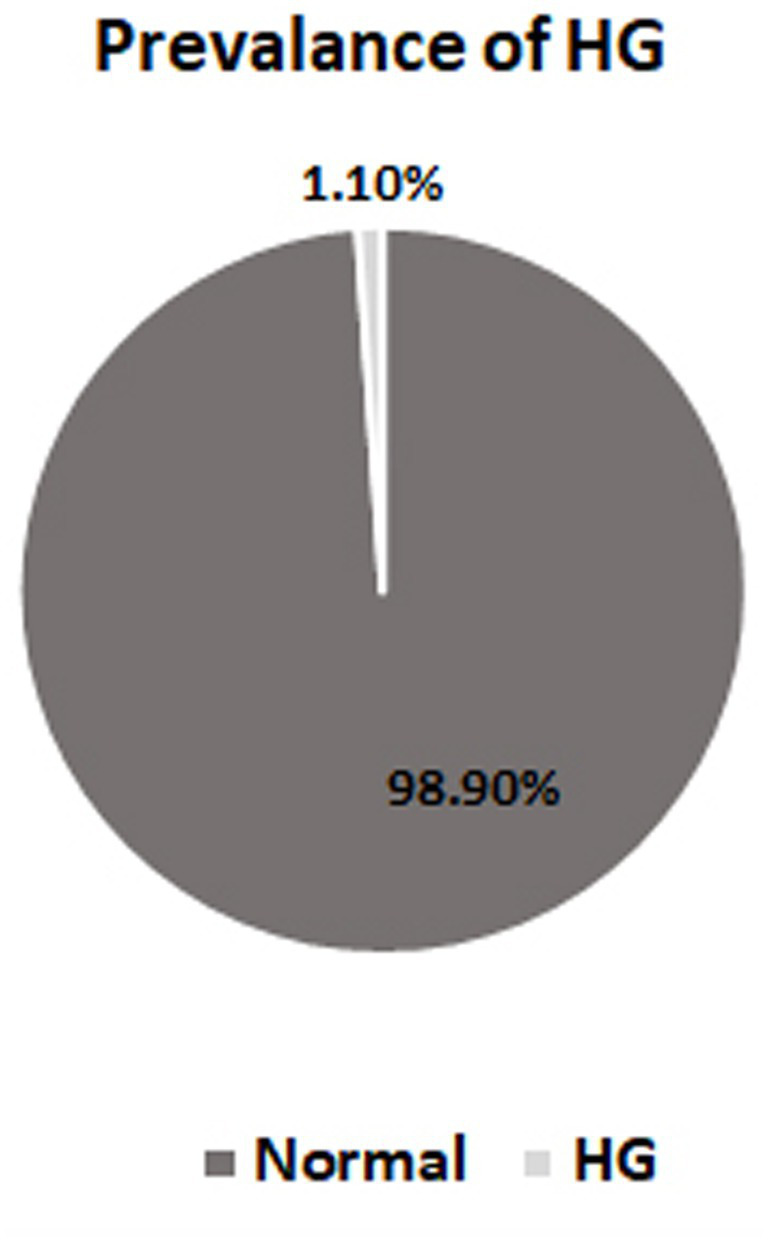
Prevalence of hyperemesis gravidarum cases. HG, hyperemesis gravidarum.

The prevalence of HG cases was 1.1% of the total 9,090 medical records reviewed from November 2020 to November 2023. These records belonged to pregnant women who received antenatal care or were admitted to the Maternity and Children Hospital in Sakaka, Aljouf, Saudi Arabia (Figure 1).

### 3.2 Demographic, obstetric, and medical data

The mean maternal age in HG cases was lower (29.03 years old) than that in control cases. The mean body weights for the HG cases were significantly lower (74.2 kg) than those for the control cases. Moreover, the mean BMI for HG cases was significantly lower (21.7) than that of control cases, as shown in Table 1. The distribution of age among the cases studied demonstrated that 4% of the HG cases were in individuals younger than 20 years, 43% were between 20 and 29 years, 48% were in the 30 to 39 age range, and 5% of HG cases were 40 years old or more (Table 2).

**Table 1:** Demographic and medical data of HG cases and controls.

Character	Control group Mean ±SD	HG group Mean ±SD	*T* value	*p*-value
Maternal age (years)	31.01 ± 5.8	29.03 ± 6.0	1.97	0.05
Body weight (kg)	80.3 ± 14.37	74.2 ± 12.24	3.23	<0.001*
BMI	28.9 ± 4.9	21.7 ± 4.1	11.27	<0.001*

*p* < 0.05 is statistically significant; analysis was done by an independent *t*-test. * represents a significant result.

**Table 2:** Age group frequency in HG cases and controls.

Character	Control group No. (%)	HG group No. (%)	Test value *χ*^2^	*p*-value
Less than 20	2 (2%)	4 (4%)	0.69	0.68
20–29	34 (34%)	43 (43%)	1.71	0.24
30–39	53 (53%)	48 (48%)	0.50	0.57
40 or more	11 (11%)	5 (5%)	2.45	0.19

*p* < 0.05 is statistically significant; analysis was done by chi-square test. * represents a significant result. *p*-value is confirmed by the exact Fisher’s test *p*-value for small numbers.

The mean gestational age for HG cases was significantly lower (15.6 weeks) than that for control cases (Table 3). This gestational age for HG cases was assessed at the time of admission to the hospital when they first presented with symptoms and at the time of the analysis of the medical records, whereas the mean gestational age for controls was estimated at the time of the analysis of the medical records during their antenatal visit.

**Table 3:** Obstetric data of HG cases and controls.

Character	Control group	HG group	Test value	*p*-value
	Mean ±SD	Mean ±SD	*T* value	
Gestational age (weeks)	17.8 ± 1.1	15.6 ± 3.7	5.7	<0.001*
	No. (%)	No. (%)	*χ* ^2^	
Gestational semester (first trimester)	18 (18%)	52 (52%)	25.41	<0.001*
Gravidity (primigravida)	15 (15%)	36 (36%)	11.61	<0.001*
Type of gestation (multiple pregnancy)	5 (5%)	11 (11%)	2.44	0.03*
History (for multigravida)				
Abortion	3 (3.53%)	5 (7.81%)	1.32	0.25
Previous HG	0 (0%)	7 (10.94%)	9.75	0.002*

*p* < 0.05 is statistically significant; analysis was done by an independent *t*-test and chi-square test. * Represents a significant result. *p*-value is confirmed by the exact Fisher’s test *p*-value for small numbers.

Table 3 shows that 52% of HG cases were in the first trimester, and the remaining cases were at the beginning of the second trimester. None of the cases exceeded 20 weeks of gestation, as reported in the medical records at the time of hospital admission and at the time of the analysis. This was significantly higher than the control group (*p*-value < 0.001); 36% of HG cases were primigravida, which was also significantly higher than controls (*p*-value < 0.001); 11% of HG cases involved multiple pregnancies, with a significant *p*-value of 0.03 when compared to the controls; 7.81% of HG cases had a previous history of abortion; and 10.94% of HG cases had a previous history of HG with a significant *p*-value of 0.002 when considering only the multigravida cases in comparison with the controls.

### 3.3 Analysis of the risk factors for HG

Table 4 presents the results of the multivariate logistic regression analysis of risk factors for HG. Binary logistic regression analysis showed that reduced maternal age emerged as the most significant predictor [Crude Odds Ratio (COR) = 0.936, 95% CI: 0.892–0.938, *p* = 0.008]. Reduced maternal body weight was a notable risk factor, with a decrease in maternal body weight elevating the risk of the outcome (COR = 0.930 per kg, 95% CI: 0.908–00953, *p* < 0.001). Correspondingly, reduced gestational age heightened the risk of HG (COR = 0.752 per week, 95% CI: 0.683–0.828, *p* < 0.001). In addition, first trimester and primigravida represented significant risk factors for HG (COR = 0.203, 0.314, *p* = <0.001, <0.01, respectively). After adjusting for potential confounders in the multivariable binary logistic regression analysis, reduced maternal body weight, gestational age, and first trimester [Adjusted Odds Ratio (AOR) = 0.913, 0.717, 0.132, *p* = <0.001, <0.001, 0.016, respectively] were significantly associated with HG.

**Table 4:** Multivariable logistic regression analysis of risk factors for hyperemesis gravidarum.

Predictor (s)	Simple logistic regression				Multiple logistic regression^a^		
	Regression coefficient (B)	Crude odds ratio (COR) (95% CI)	Wald statistic	*p*-value	Regression coefficient (B)	Adjusted odds ratio (AOR) (95% CI)	*p*-value
Maternal age (years)	−0.066	0.936 (0.892–0.983)	7.133	0.008^*^	−0.005	1.005 (0.942–1.072)	0.888
Body weight (kg)	−0.072	0.930 (0.908–0.953)	33.728	<0.001^*^	−0.091	0.913 (0.883–0.944)	<0.001^*^
Gestational age (weeks)	−0.285	0.752 (0.683–0.828)	33.463	<0.001^*^	−0.333	0.717 (0.633–0.812)	<0.001^*^
Gestational semester (first trimester)							
No	0	1			0	1	
Yes	1.596	0.203 (0.106–0.386)	23.637	<0.001^*^	2.027	0.132 (0.025–0.685)	0.016^*^
Primigravida							
No	0	1			0	1	
Yes	1.159	0.314 (0.158–0.622)	11.030	<0.01^*^	0.205	1.228 (0.195–7.748)	0.827
Multiple pregnancy							
No	0	1			0	1	
Yes	0.854	0.426 (0.142–1.274)	2.331	0.127	0.142	0.868 (0.143–5.245)	0.877

a Multiple logistic regression model was applied.

*p-value < 0.05 = statistically significant.

COR, crude odds ratio; AOR, adjusted odds ratio.

Maternal age, primigravida status, and multiple gestations were not substantially correlated with the results of this study. Variables with extremely low event counts, such as a history of HG and a history of abortion, were excluded from the model due to their contribution to unstable coefficient estimates with high standard errors, which rendered the results unreliable.

### 3.4 Analysis of the main consequences of HG

#### 3.4.1 Blood pressure measurements

**Figure 2:**
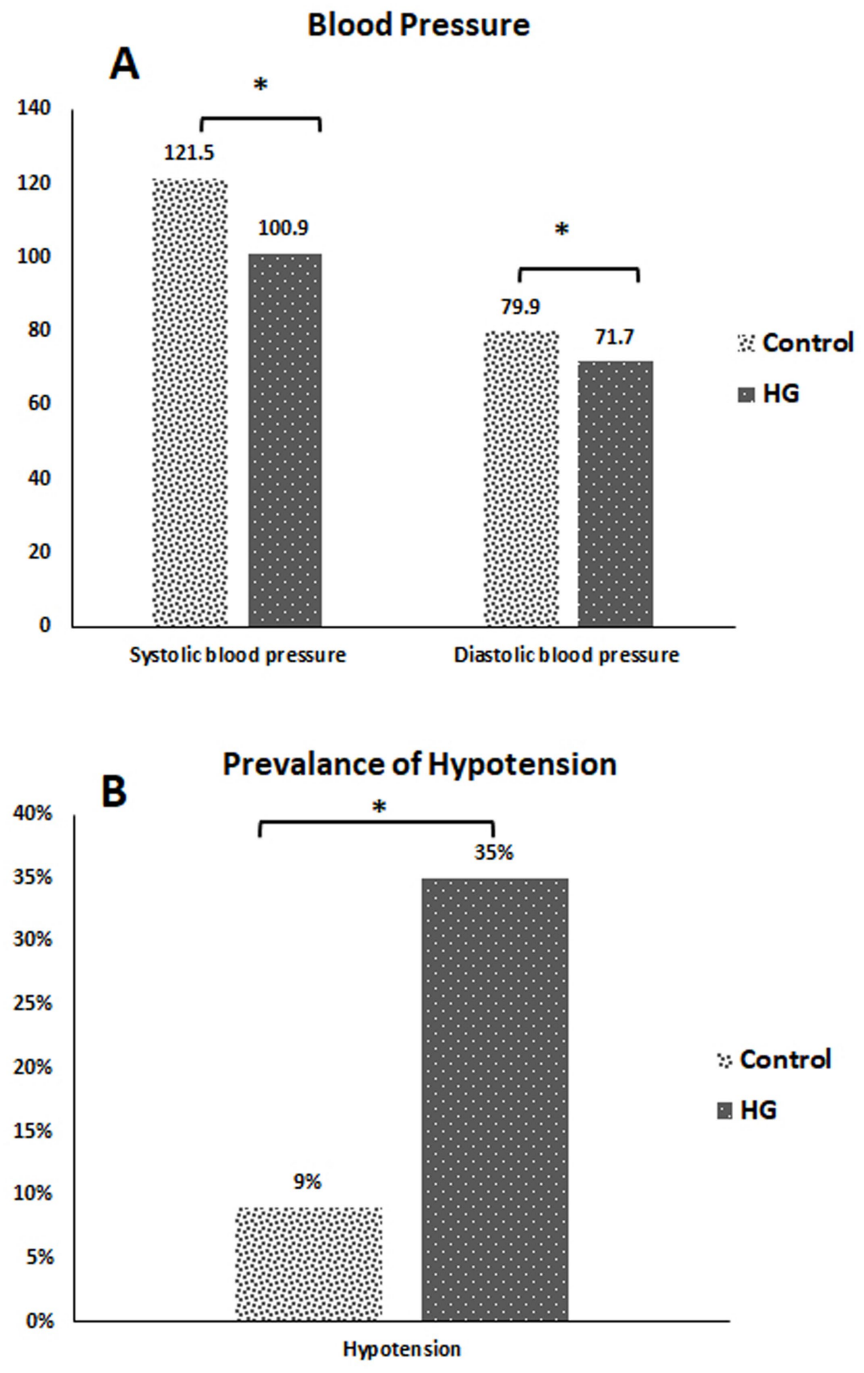
**(A)** Systolic and diastolic blood Pressure for the studied cases. **(B)** Prevalence of hypotension among the studied cases. *p-value < 0.05 = statistically significant. HG, hyperemesis gravidarum.

The mean systolic blood pressure for HG cases was significantly lower (100.9 mmHg) than that for control cases. The mean diastolic blood pressure for HG cases was significantly lower (71.7 mmHg) than that for control cases (Figure 2A). Hypotension was observed in 35% of the HG cases (Figure 2B).

#### 3.4.2 Serum electrolyte assay results

**Figure 3:**
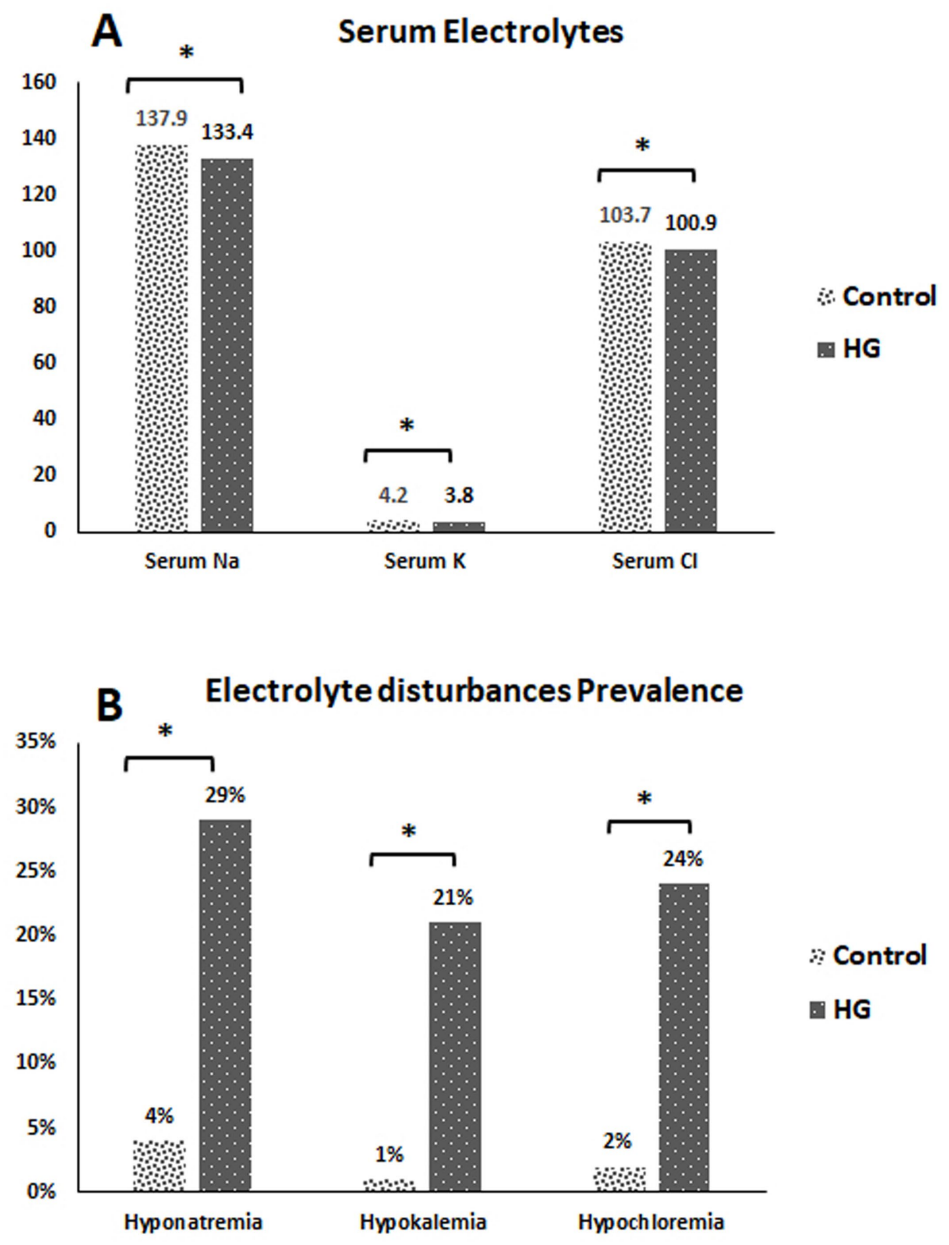
**(A)** Serum electrolyte disturbance for the studied cases. **(B)** Prevalence of electrolyte disturbance among the studied cases. *p-value < 0.05 = statistically significant. HG, hyperemesis gravidarum.

The mean serum Na in HG cases was significantly lower (133.4 mEq/L) compared to control cases. Additionally, the mean serum K in HG cases was significantly lower (3.8 mEq/L) compared to controls. Moreover, the mean serum Cl in HG cases was significantly lower (100.9 mEq/L) compared to controls (Figure 3A). Furthermore, 29% of HG cases had hyponatremia, 21% had hypokalemia, and 24% had hypochloremia (Figure 3B).

#### 3.4.3 Complete blood count results

**Figure 4:**
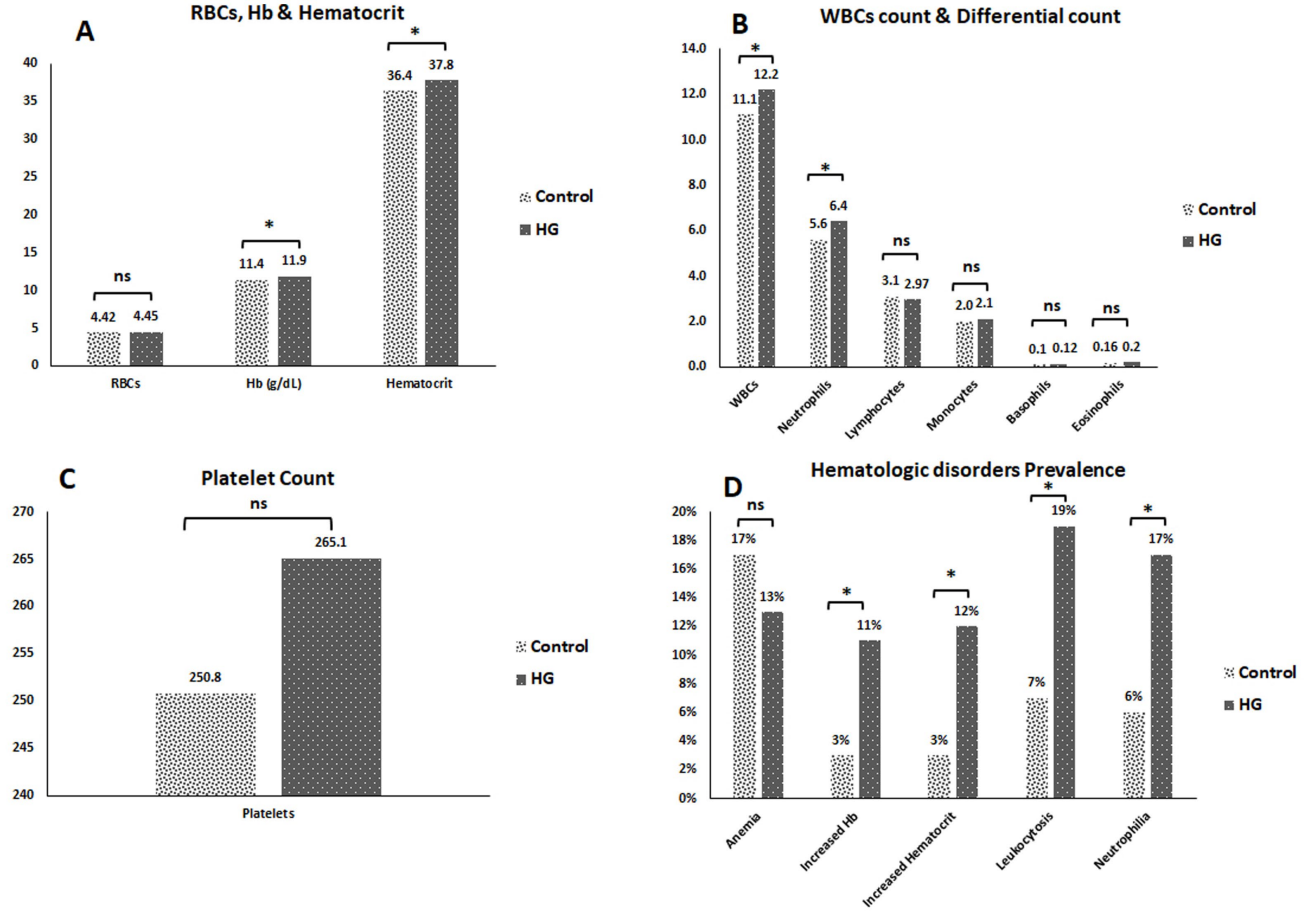
**(A)** RBCs count, Hb concentration & haematocrit for the studied cases. **(B)** WBCs count & the differential WBCs count for the studied cases. **(C)** Platelets count for the studied cases. **(D)** Prevalence of hematologic disturbance among the studied cases. *p-value < 0.05 = statistically significant. ns, non-significant; HG, hyperemesis gravidarum; RBCs, red blood cells; Hb, hemoglobin; WBCs, white blood cells.

The mean RBC count for HG cases, was not significantly higher (4.45 /mm^3^ × 106) compared to control cases. The mean Hb levels for HG cases was significantly increased (11.9 g/dL) than that for controls. Additionally, the mean hematocrit level for HG cases was significantly higher (37.8%) compared to controls. The mean WBC count for HG cases was significantly higher (12.2/mm^3^ × 10^3^) than that for the control cases. For WBC differential count, the neutrophil count was significantly increased (6.4/mm^3^ × 10^3^) in HG cases compared to controls. The lymphocyte count was insignificantly lower (2.97/mm^3^ × 10^3^) in HG cases than in controls. The monocyte count was insignificantly higher (2.1/mm^3^ × 10^3^) in HG cases than in controls. Similarly, the basophil count was insignificantly higher (0.12/mm^3^ × 103) in HG cases than in controls. The eosinophil count was also insignificantly increased (0.2/mm^3^ × 10^3^) in HG cases compared controls. The mean platelet count in HG cases was insignificantly higher (265.1 /mm^3^ × 10^3^) than that in control cases (Figures 4A–C).

Figure 4D demonstrated that 13% of HG cases had anemia, 11% of HG cases had an increased Hb level, 12% had increased hematocrit level, 19% of HG cases had leukocytosis and 17% had neutrophilia.

## 4 Discussion

The increased prevalence of HG is a global public health concern. It is a severe and persistent complication of early pregnancy that may require hospitalization (4). Our study focused on the assessment of the prevalence of hyperemesis gravidarum among pregnant women in the Aljouf Region and the most important risk factors of this condition.

The findings of this study revealed that the prevalence of HG was 1.1% of the total number of pregnant women examined. This prevalence aligns with previous studies that demonstrated an incidence of HG ranging from 0.30 to 3.6% of pregnancies (2).

According to the findings of the current study, the age distribution among the cases studied ranged from 20 to 29 years old to 3–-39 years old. The body weights and BMI of HG cases were significantly lower than those of the controls. The first trimester and low gestational age were the most significant risk factors for HG. This was consistent with a previous study that clarified that pregnant women in the age group of 20–24 years and underweight were associated with severe hyperemesis gravidarum (14).

Furthermore, Schiff et al. (15) mentioned that the majority of women with HG (86.4%) were in the 20–35 age group. Additionally, HG cases showed more than 5% pre-pregnancy weight loss and dehydration. In pregnancies affected by HG, inadequate total maternal weight gain and failure to regain pre-pregnancy weight by weeks 13–18 are associated with being small for gestational age (16).

The current study showed that 36% of HG cases were primigravida. This finding is in line with a previous study that reported that 36.2% of people with an HG diagnosis were primigravida. HG was found to be substantially correlated with primigravidity, possibly due to stress and first-time exposure to elevated HG levels (17). Additionally, the majority of women with HG were admitted during the first trimester, which is in line with the findings of an earlier study (18). Furthermore, the average gestational age reported in the study by David et al. (19) was 9.8 weeks.

The present study found that 11% of HG cases had multiple pregnancies. Similarly, a previous study found that twin cases accounted for 4.7% of HG cases and 2.4% of control cases (20). This can be explained by the fact that women with multiple pregnancies have markedly elevated levels of HG hormones that aggravate vomiting during pregnancy (21).

Similarly, the current results revealed that 7.81% of HG cases had a history of abortion and 10.94% had a history of HG. According to a previous study, women with an abortion history had a sixfold higher chance of HG than those without an abortion history (22). In addition, another study found that women with a history of HG had an 11-fold increased risk of developing HG compared to their counterparts (23).

This study found that one of the primary consequences of HG was hypotension. This was consistent with the study’s conclusion that malfunction of the autonomic nervous system, which led to hypotension, was the cause of notable blood pressure fluctuations. The low blood pressure readings may have been caused by the frequent vomiting episodes associated with HG, which led to severe hypovolemia and worsened postural hypotension due to autonomic dysfunction (24).

The current results showed that serum Na, K, and Cl levels in HG cases were significantly lower than those in controls, which is considered an important consequence of HG. According to the results of an earlier study, almost 40% of women with HG had hypokalemia, whereas 36 and 8% of those women had hyponatremia and hypochloremia, respectively (11). Furthermore, a previous study verified that HG is sufficiently toxic to cause hypokalemia, acidosis from fasting, weight loss, dehydration, and alkalosis from vomiting hydrochloric acid (25). The severity of hypokalemia caused by HG may be accelerated by abnormal fluid loss through vomiting, inadequate intake, and incorrect fluid infusion (26).

HG can also cause hyponatremia. Anorexia, headache, nausea, vomiting, and fatigue are some of the clinical signs of mild hyponatremia that might be difficult to differentiate from hyperemesis. Personality changes, muscle weakness and cramping, ataxia, confusion, lethargy, reduced reflexes, and convulsions can all be symptoms of more severe hyponatremia (27).

The present data demonstrated that the RBC count in HG cases was not significantly higher than that in the controls. Similarly, an earlier study discovered that there was no discernible difference in RBC count between HG patients and controls, and our findings were in line with this result (28).

Furthermore, the Hb and hematocrit levels for HG cases were significantly higher compared to control cases. This was consistent with a previous study that discovered that patients with HG had elevated levels of hemoglobin and hematocrit. Excessive vomiting, fluid loss, and dehydration may lead to hemoconcentration and increased Hb and hematocrit levels (29).

The platelet count in HG cases was insignificantly higher compared to controls. Similarly, a recent study found no significant difference in platelet count between HG patients and controls, suggesting that platelet activation is not the pathogenetic mechanism underlying HG (30).

The current study discovered that the WBC count for HG cases was significantly higher compared to control cases. Regarding WBC differential count, the neutrophil count was significantly increased in HG cases, while the lymphocyte count was insignificantly decreased in HG cases. Similarly, a previous study emphasized that patients with HG had significantly higher serum levels of markers of acute and chronic inflammation, such as oxidative stress, and inflammation is a key component in the pathogenesis of HG. Thus, pregnant HG patients had significantly higher serum levels of WBCs and neutrophils than healthy controls (31).

## 5 Conclusion

Hyperemesis gravidarum (HG) is a condition that commences early in pregnancy, usually before 16 weeks of gestation, and is marked by severe nausea and/or vomiting and an inability to consume food and/or liquids normally. This condition can lead to weight loss, electrolyte imbalance, and hematologic disturbance. This study concludes that being in the first trimester of pregnancy and having a low gestational age are the primary risk factors for HG. Therefore, HG should be considered by medical professionals during the initial prenatal care visit.

### 5.1 Strengths of the study

The current study is the first of its kind on HG in the Al-Jouf region of Saudi Arabia, highlighting the prevalence of this important health issue. This study thoroughly characterizes the demographic, clinical, and obstetrical features of affected women, offering valuable insights into the risk factors and outcomes of HG in this region. These results have obvious implications for clinical practice, guiding early detection, risk assessment, and customized management methods in local healthcare centers. This study not only deepens our understanding of HG in the Al-Jouf region but also encourages strategies aimed at enhancing maternity care and health outcomes and paves the way for further research.

### 5.2 Limitations of the study

This research was limited by the availability of accessible medical records and the scope of investigations within them, which is intrinsic to its retrospective design. A selection bias may have affected the inclusion and exclusion criteria. Moreover, assessments of disease severity were not uniformly accessible. The absence of information regarding pregnancy outcomes limits the comprehensive evaluation of the clinical impact of HG.

## Data Availability

The original contributions presented in the study are included in the article/supplementary material, further inquiries can be directed to the corresponding author.
